# Three J-proteins impact Hsp104-mediated variant-specific prion elimination: a new critical role for a low-complexity domain

**DOI:** 10.1007/s00294-019-01006-5

**Published:** 2019-06-22

**Authors:** Scott E. Berger, Anna M. Nolte, Erina Kamiya, Justin K. Hines

**Affiliations:** 1 Department of Chemistry, Lafayette College, Easton, PA 18042 USA; 2 Present Address: Optical Microscopy and Analysis Laboratory, Office of Science and Technology Resources, Center for Cancer Research, National Cancer Institute, National Institutes of Health, Frederick, MD 21702 USA

**Keywords:** Prion, Molecular chaperone, Hsp104, Protein misfolding, Yeast

## Abstract

Prions are self-propagating protein isoforms that are typically amyloid. In *Saccharomyces cerevisiae*, amyloid prion aggregates are fragmented by a trio involving three classes of chaperone proteins: Hsp40s, also known as J-proteins, Hsp70s, and Hsp104. Hsp104, the sole Hsp100-class disaggregase in yeast, along with the Hsp70 Ssa and the J-protein Sis1, is required for the propagation of all known amyloid yeast prions. However, when Hsp104 is ectopically overexpressed, only the prion [*PSI*^+^] is efficiently eliminated from cell populations via a highly debated mechanism that also requires Sis1. Recently, we reported roles for two additional J-proteins, Apj1 and Ydj1, in this process. Deletion of Apj1, a J-protein involved in the degradation of sumoylated proteins, partially blocks Hsp104-mediated [*PSI*^+^] elimination. Apj1 and Sis1 were found to have overlapping functions, as overexpression of one compensates for loss of function of the other. In addition, overexpression of Ydj1, the most abundant J-protein in the yeast cytosol, completely blocks Hsp104-mediated curing. Yeast prions exhibit structural polymorphisms known as “variants”; most intriguingly, these J-protein effects were only observed for strong variants, suggesting variant-specific mechanisms. Here, we review these results and present new data resolving the domains of Apj1 responsible, specifically implicating the involvement of Apj1’s Q/S-rich low-complexity domain.

## Introduction

Prions are self-propagating transmissible agents that are typically comprised of highly ordered and thermodynamically stable amyloid protein aggregates (Liebman and Chernoff 2012). Typically, the phenotype of a prion is a partial loss-of-function of the prionogenic protein. Some prion-forming primary sequences have been shown to form multiple distinct structural polymorphisms that influence the severity of prion-associated phenotypes; these are called “strains” in mammals and “variants” in yeast (Derkatch et al. 1996; Tanaka et al. 2006; Liebman and Chernoff 2012; Prusiner 2013).

Yeast prions require molecular chaperone activity for stable propagation in cell populations (Tuite and Koloteva-Levin 2004; Liebman and Chernoff 2012). To date, 12 prions have been identified in *Saccharomyces cerevisiae*. Of these, 10 are amyloid-based (Kondrashkina et al. 2014; Wickner et al. 2015). Current models assert that Hsp40-class chaperones, which are cochaperones to Hsp70s, recognize and recruit Hsp70 to the amyloid fibril. Hsp70 then recruits Hsp104, the chaperone responsible for the physical severing of amyloid fibrils (Cox et al. 2003; Aron et al. 2007; Higurashi et al. 2008; Tipton et al. 2008; Winkler et al. 2012; Harris et al. 2014). Hsp104, which plays a critical role in the solubilization of stress-induced protein aggregates (Wendler et al. 2009; Yokom et al. 2017), is necessary for the propagation of all known amyloid-based prions (Wickner et al. 2018). Hsp104 threads prion monomers from amyloid cores through its central cavity, solubilizing them and eventually remodeling and ultimately fragmenting the amyloid aggregates to generate new propagons—the unit of proteinaceous infection particles that must be propagated to maintain the prion state (Cox et al. 2003; Haslberger et al. 2008; Tipton et al. 2008; Winkler et al. 2012).

Hsp70s are a versatile class of molecular chaperones that are essential for a variety of cellular functions including protein folding and transport, iron–sulfur cluster biogenesis, liquid-drop aggregation, and notably, prion propagation (Jung et al. 2000; Kampinga and Craig 2010; Craig and Marszalek 2017; Simpson-Lavy and Kupiec 2018). The ability of Hsp70s to fine-tune their functions stems from their biochemical activity; they engage in a promiscuous and ATP-dependent client-binding and release cycle that is directed, enhanced, and calibrated by Hsp40s and nucleotide exchange factors (NEFs) (Kampinga and Craig 2010; Craig and Marszalek 2017; Schilke et al. 2017). Cochaperone Hsp40s (also and hereafter called “J-proteins”) stimulate Hsp70 ATPase activity via their characteristic J-domains (Li et al. 2009; Craig and Marszalek 2017). In *S. cerevisiae*, there are 13 J-proteins that at least temporarily occupy the cytosol. One of these J-proteins, Sis1, is essential for both cell survival and propagation of the four best-studied yeast prions: [*PSI*^+^], [*RNQ*^+^], [*URE3*], and [*SWI*^+^] (Luke et al. 1991; Sondheimer et al. 2001; Aron et al. 2007; Higurashi et al. 2008; Hines et al. 2011b). Specific chaperone requirements for propagation are largely differentiable among different prions and prion variants, particularly with respect to domain requirements of J-proteins (Derkatch et al. 1996; Hines et al. 2011a; Prusiner 2013; Stein and True 2014; Sporn and Hines 2015; Schilke et al. 2017; Killian and Hines 2018; Killian et al. 2019). In addition to Sis1, three other J-proteins—Ydj1, Swa2, and Apj1—have been previously implicated in prion biology. Ydj1 is the most abundant J-protein in the yeast cytosol and is required in addition to Sis1 for propagation of the prion [*SWI*^+^] (Ghaemmaghami et al. 2003; Hines et al. 2011b), whereas Swa2 is required in addition to Sis1 for the propagation of the prion [*URE3*] (Troisi et al. 2015; Oliver et al. 2017). Apj1, a J-protein similar in structure to Ydj1, is involved in the degradation of sumoylated proteins and has been demonstrated to eliminate synthetic prions when overexpressed (hence its name, “anti-prion DnaJ”) (Kryndushkin et al. 2002; Sahi et al. 2013).

**Fig. 1:**
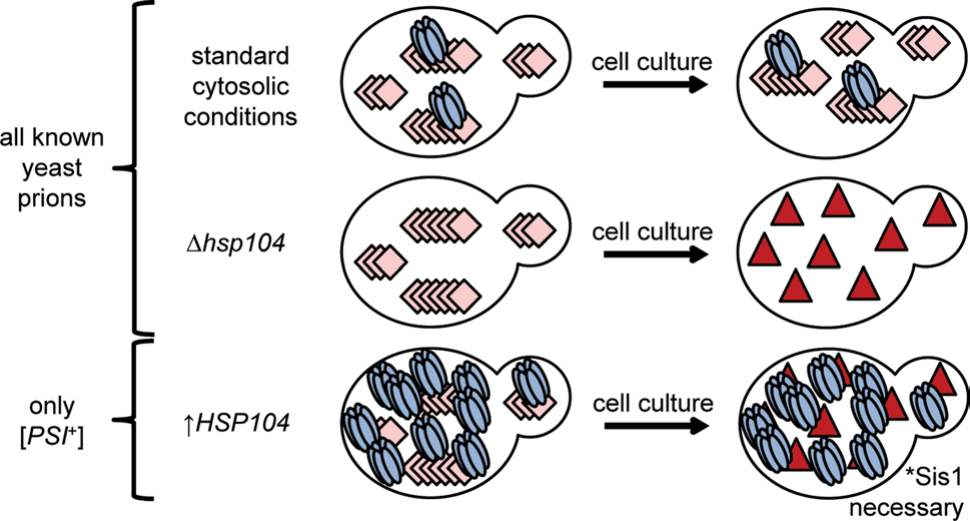
Effect of Hsp104 expression on yeast prion propagation. Pink diamonds represent amyloid fibers, and red triangles represent soluble prionogenic protein. Deletion of the *HSP104* gene results in the loss of all amyloid yeast prions, as does chemical inhibition of Hsp104 ATPase activity by guanidine hydrochloride. Overexpression of Hsp104 destabilizes [*URE3*] and results in the efficient curing of the prion [*PSI*^+^], but not other prions (Matveenko et al. 2018)

Ectopic overexpression of Hsp104 destabilizes [*URE3*] but efficiently eliminates only the prion [*PSI*^+^] (Fig. 1) (Chernoff et al. 1995; Derkatch et al. 1997; Moriyama et al. 2000; Volkov et al. 2002; Du et al. 2008; Patel et al. 2009; Saifitdinova et al. 2010; Holmes et al. 2013; Matveenko et al. 2018). The mechanism of Hsp104-mediated elimination of [*PSI*^+^] is the subject of significant current debate as several contradictory models have been proposed in the recent literature (Helsen and Glover 2012; Winkler et al. 2012; Park et al. 2014; Zhao et al. 2017; Ness et al. 2017; Cox and Tuite 2018; Matveenko et al. 2018). Here, we will revisit our recent findings (Astor et al. 2018) and present some additional data on the roles of J-proteins in Hsp104-mediated elimination of [*PSI*^+^]. Considering our new findings, we expand on our understanding of the role of Apj1 in this process and underscore the importance of low-complexity regions in prion biology.

## The role of Sis1 in Hsp104-mediated curing of [*PSI*^+^]

The human ortholog of Sis1, Hdj1, is sufficient in substituting for Sis1 in maintenance of cell viability and [*RNQ*^+^] propagation when highly expressed (Lopez et al. 2003). Hdj1 is also sufficient in substituting for Sis1 in the propagation of strong, but not weak variants of [*PSI*^+^] (Harris et al. 2014), although it is insufficient in Hsp104-mediated elimination of [*PSI*^+^] (Sporn and Hines 2015). In continuing this line of investigation, we recently showed that the Sis1 *Drosophila melanogaster* ortholog Droj1 supports the propagation of strong, but not weak [*PSI*^+^] variants, and like Hdj1, Droj1 was also deficient in supporting Hsp104-mediated [*PSI*^+^] elimination (Astor et al. 2018).

**Fig. 2:**
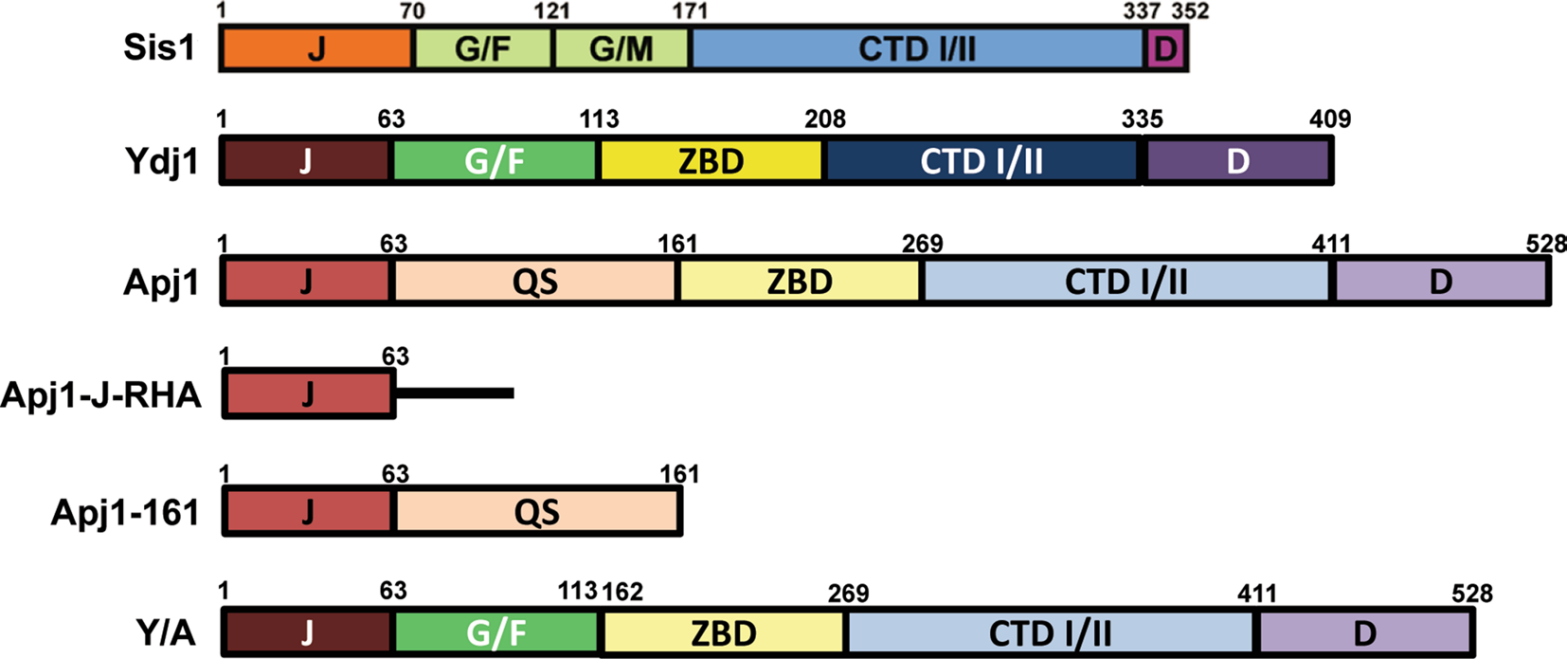
J-protein primary structure diagrams. Summary of primary sequences of J-proteins and J-protein constructs used in this study. Protein regions are denoted as follows: *J* J-domain, *G/F* glycine/phenylalanine-rich region, *G/M* glycine/methionine-rich region, *QS* glycine-rich region also rich in glutamine and serine, *ZBD* zinc-binding domain, *CTD I/II* C-terminal peptide-binding domains I and II, *D* dimerization domain. Black line represents random peptide sequence with a C-terminal human influenza hemagglutinin (HA) tag

To further investigate the role of Sis1 in Hsp104-mediated curing of [*PSI*^+^], we used two previously characterized constructs that are sufficient in stably propagating [*PSI*^+^] (Harris et al. 2014): Sis1ΔG/F, which lacks the glycine and phenylalanine-rich region, and Sis1-121, a construct solely comprised of the J-domain and G/F region (see Fig. 2 for Sis1 domain structure). Sis1ΔG/F can support the propagation of both strong and weak variants of [*PSI*^+^] and, interestingly, it also supports Hsp104-mediated [*PSI*^+^] curing of a weak, but not a strong variant. Sis1-121 is unable to support propagation of weak [*PSI*^+^] (Harris et al. 2014) and, like Sis1ΔG/F, it is deficient in its ability to support Hsp104-mediated curing of a strong [*PSI*^+^] variant. Therefore, we concluded that Sis1’s activity in [*PSI*^+^] propagation and Hsp104-mediated curing of strong [*PSI*^+^] are mechanistically distinct, as both the Sis1 constructs and orthologs selectively permit only one of the two processes. In addition, this suggests that Hsp104-mediated [*PSI*^+^] curing may be variant-specific, as Sis1ΔG/F was able to support the curing of weak, but not strong [*PSI*^+^].

## Sis1, Apj1, and Ydj1 all profoundly affect elimination of strong [*PSI*^+^] variants

Prior to our investigation, four cytosolic J-proteins were implicated in prion biology: Sis1, Apj1, Swa2, and Ydj1 (Kryndushkin et al. 2002; Bradley et al. 2002; Lian et al. 2007; Hines et al. 2011a; Hines and Craig 2011; Troisi et al. 2015; Verma et al. 2017; Oliver et al. 2017; Killian and Hines 2018). Given 13 J-proteins at least partially inhabit the yeast cytosol (Sahi and Craig 2007) and three (Sis1, Ydj1, and Swa2) have been found to play critical roles in prion propagation, we hypothesized that other J-proteins may be involved in Hsp104-mediated curing of [*PSI*^+^]. As a first step toward testing this hypothesis, we utilized a screen with strains lacking each J-protein individually and determined that no J-protein other than Sis1 is required for the stable propagation of two different and well-studied weak [*PSI*^+^] variants: [*PSI*^+^]^Sc37^ and [*PSI*^+^]^VL^. We then utilized this same set of J-protein deletion strains to determine if any of these 12 other J-proteins are required for efficient Hsp104-mediated [*PSI*^+^] elimination. This screen revealed that in addition to Sis1, Apj1 is also required for efficient Hsp104-mediated curing, but only for the strong variants; no evidence was uncovered that any J-protein, including Sis1, is necessary for the elimination of weak [*PSI*^+^] variants (Astor et al. 2018).

We next explored whether Sis1 and Apj1 may have overlapping functions. Indeed, we found that Hsp104-mediated curing of strong variants is restored when Apj1 is overexpressed in a [Sis1ΔG/F], Δ*sis1* background. Likewise, the overexpression of Sis1 in a Δ*apj1* background restores efficient curing. Since Sis1 is known to accelerate curing when overexpressed (Kryndushkin et al. 2011), we also wondered if Apj1 would behave similarly given its overlapping function with Sis1. As predicted, overexpression of either protein independently accelerates curing of a strong [*PSI*^+^] variant, which led us to test whether the overexpression of any other J-protein might also accelerate curing. An attempt to use Ydj1 for this purpose then led to the surprising discovery that Ydj1 overexpression potently blocks Hsp104-mediated curing, and this effect is specific to strong [*PSI*^+^] variants. We then confirmed this effect was not due to a peculiarity of a specific strong prion variant by running identical experiments involving Sis1, Apj1, and Ydj1 using a second strong variant. Finally, we tested whether both alterations that block strong [*PSI*^+^] curing, when combined, could affect weak [*PSI*^+^]. Strikingly, cells simultaneously lacking Apj1 and overexpressing Ydj1 were still cured of weak [*PSI*^+^] by Hsp104 overexpression, consistent with the hypothesis that the mechanisms by which weak and strong variants are eliminated by Hsp104 may be biochemically distinct.

## Apj1 domain analysis

Our recent findings raise the question of what specific domains of Apj1 are required for its function in Hsp104-mediated elimination of strong [*PSI*^+^] variants, which we were unable to address in our original investigation but will address here. To achieve domain-level resolution, we tested constructs of Apj1 that are known to be functional and significantly expressed in vivo in two strains bearing the strong variants [*PSI*^+^]^STR^ and [*PSI*^+^]^Sc4^. The domain architectures of the constructs used in this experiment as well as those of Sis1 and Ydj1 are shown in Fig. 2.

**Fig. 3:**
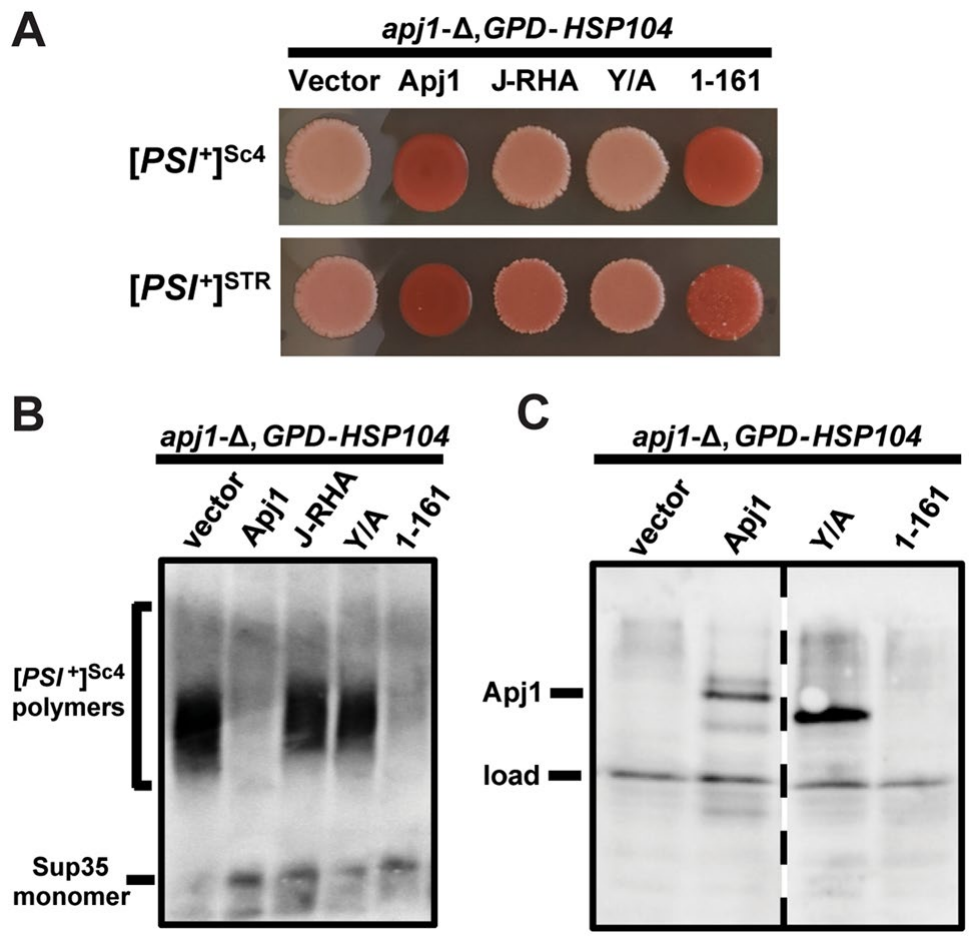
Apj1 domain analysis for Hsp104-mediated elimination of strong [*PSI*^+^] variants. **a** Strong [*PSI*^+^]^STR^ and [*PSI*^+^]^Sc4^ cells of the W303 genetic background lacking genomic *APJ1* were transformed with plasmids expressing wild-type Apj1, Apj1-J-RHA, the first 161 residues of Apj1, or the Y/A chimera of Ydj1 and Apj1. Cells were then transformed with a plasmid overexpressing Hsp104 (*GPD*-*HSP104*) that normally cures [*PSI*^+^]. Color phenotype assays are shown for representative transformants (*n* = 10 for each variant). **b** Lysates of representative cells from panel A were resolved by SDD-AGE and subjected to immunoblot analysis using polyclonal antibody raised against Sup35. **c** Lysates of representative cells from panel A were resolved by SDS-PAGE and subjected to immunoblot analysis using polyclonal antibody raised against Apj1. Load control shown is a nonspecific protein cross-reacting with our Apj1 primary antibody

Plasmids expressing Apj1 constructs (Table 1) or an empty vector were used to transform Δ*apj1* cells of the W303 genetic background bearing one of two strong [*PSI*^+^] variants. Transformants were grown on selective medium at 30 °C for 2 days and then cells were subsequently transformed with either another empty vector control (not shown) or a plasmid overexpressing Hsp104. 10 transformants were plated to selective solid medium and repatched twice at 30 °C, then were subsequently transferred to YPD medium to develop color (Fig. 3a). In all cases, strains which received the second empty vector, and, therefore, lacked Hsp104 overexpression, maintained [*PSI*^+^] as expected (data not shown). As originally reported in Astor et al. 2018, we again confirmed that the deletion of Apj1 greatly reduces or completely eliminates curing (Fig. 3a, first column; [*PSI*^+^]^STR^: 0/10 cured, [*PSI*^+^]^Sc4^: 0/10 cured) and again subsequently reconfirmed that Apj1 is specifically responsible for this effect through the observed restoration of curing following restoration of Apj1 expression (Fig. 3a, second column; [*PSI*^+^]^STR^: 10/10 cured, [*PSI*^+^]^Sc4^: 9/10 cured). Since the J-domain is critical for the simulation of Hsp70 ATPase activity (Tsai and Douglas 1996), we next determined if Apj1’s J-domain alone was sufficient in carrying out its role in this process. To do this, we used a construct in which the J-domain of Apj1 is fused with a C-terminal 10 residue random linker and a C-terminal HA tag (Apj1-J-RHA, Fig. 2); this construct is functional and expressed at a high enough level to rescue the growth phenotype of Δ*ydj1* cells (Sahi and Craig 2007) as well as to cure the prion [*SWI*^+^] (Hines et al. 2011b). Despite this, Apj1-J-RHA was not sufficient to replace Apj1 (Fig. 3a; [*PSI*^+^]^STR^: 1/10 cured, [*PSI*^+^]^Sc4^: 0/10 cured).

**Table 1:** Plasmids used in this study

Plasmid	Marker	Copy number	Source
pRS424GPD-APJ1	TRP	2μ, high	Sahi and Craig (2007)
pRS424GPD-APJ1-J-RHA	TRP	2μ, high	Sahi and Craig (2007)
pRS424GPD-APJ1_1-161_	TRP	2μ, high	Sahi and Craig (2007)
pRS414ADH1-Y/A	TRP	CEN, low	Hines et al. (2011a, b)
pRS426GPD-HSP104	URA	2μ, high	Sporn and Hines (2015)

We next postulated that the putative substrate-binding regions of Apj1 that have previously been implicated in the degradation of sumoylated proteins (Sahi et al. 2013) might be responsible for its activity in this curing process. Expressing these regions alone, however, is highly unlikely to produce a functional protein product in vivo given the critical role of the J-domain. To circumvent this technical barrier, we used the Y/A construct, a chimeric protein comprised of the J-domain and G/F region of Ydj1, followed by the zinc- and peptide-binding regions of Apj1 (Fig. 2). Ydj1-113 (only the J and G/F regions of Ydj1) is insufficient to block Hsp104 curing when overexpressed on its own (Berger and Hines, unpublished observations). The Y/A construct was chosen for this reason, and because it is functional in vivo and expressed at sufficiently high levels to rescue the Δ*ydj1* growth phenotype; moreover, the C-terminal domains of Apj1 were specifically able, in the context of this construct, to rescue the propagation of the [*SWI*^+^] prion in a Δ*ydj1* background, indicating that the C-terminal domains of Apj1, specifically, are functional in this construct (Hines et al. 2011b). Despite this, Y/A was also insufficient to replace Apj1 (Fig. 3a; [*PSI*^+^]^STR^: 0/10 cured, [*PSI*^+^]^Sc4^: 0/10 cured).

Finally, we tested whether a construct expressing only the J-domain and low-complexity QS region of Apj1 is sufficient (Apj1-161; Fig. 2); like the others, this construct also rescues the Δ*ydj1* phenotype (Sahi and Craig 2007). Strikingly, Apj1-161 completely restored curing to the same extent observed with the wild-type Apj1 control (Fig. 3a; [*PSI*^+^]^STR^: 10/10 cured, [*PSI*^+^]^Sc4^: 9/10 cured). To confirm that the observed changes in color correspond to the maintenance or loss of the prion rather than simply an alteration of the color phenotype, we subjected transformants shown in Fig. 3a to a biochemical assay, semidenaturing detergent agarose gel electrophoresis (SDD-AGE), in which detergent-resistant aggregates are resolved by size within an agarose gel and are then visualized by immunoblotting (Kryndushkin et al. 2003). As expected, isolates that formed pink colonies on adenine-limited medium harbored SDS-resistant Sup35 aggregates, whereas those that formed red colonies lacked Sup35 aggregates (Fig. 3b). Finally, to verify that differences in the ability to restore Apj1 function were not due to unexpected issues with protein expression, we subjected [*PSI*^+^]^STR^ cells shown in Fig. 3a to SDS-PAGE and immunoblotting analysis of representative patches using polyclonal antibody raised against Apj1. Our antibody does not recognize Apj1-J-RHA (not shown) or Apj1-161 (Fig. 3c); however, Y/A is expressed equivalently or greater than wild-type Apj1 (Fig. 3c), indicating that the absence of an effect is not due to under-expression of this construct. Likewise, we were able to exclude the possibility that the observed effect of Apj1-161 was due to unexpected expression of wild-type protein in those cells (e.g., due to homologous recombination).

## Conclusions and perspectives

To explore the potential roles of J-proteins in Hsp104-mediated curing, in Astor et al., we examined multiple [*PSI*^+^] variants and utilized well-characterized Sis1 constructs and one metazoan ortholog. We had previously shown that the human Sis1 ortholog, Hdj1, is capable of substituting for Sis1 to propagate strong, but not weak, [*PSI*^+^] variants, and that it could not replace Sis1 to promote Hsp104-mediated curing (Sporn and Hines 2015). In Astor et al., we found essentially identical results for the *D. melanogaster* ortholog, Droj1. Both of these orthologs also propagate at least some variants of the prion [*RNQ*^+^] (Lopez et al. 2003). It is worth noting that Sis1 orthologs have retained the ability to propagate some variants of [*PSI*^+^] and [*RNQ*^+^], but all higher eukaryotic orthologs examined to date lack the ability to substitute for Sis1 in Hsp104-mediated curing, now including human Hdj1 (Sporn and Hines 2015), *D. melanogaster* Droj1 (Astor et al. 2018), *A. thaliana* atDjB1 (Verma et al. 2017), and five other *A. thaliana* Sis1 orthologs (Sahi and Hines, unpublished observations). Wickner et al., have described Hsp104-mediated curing as one of many “anti-prion” functions of yeast (Wickner et al. 2015, 2018). One interpretation of these findings, in that light, might be that these orthologs have retained some prion-maintaining functions but have intriguingly lost this “anti-prion” function.

Currently, two models for Hsp104-mediated [*PSI*^+^] elimination are significantly debated in the literature: malpartitioning of prion propagons to daughter cells (Ness et al. 2017; Cox and Tuite 2018; Matveenko et al. 2018) and aggregate trimming followed by propagon degradation (Park et al. 2014; Zhao et al. 2017; Greene et al. 2018). According to the malpartitioning model, Hsp104 overexpression results in a retention of propagons in the mother cell, plausibly due to propagon attachment to the cytoskeleton (Cox and Tuite 2018). This is most clearly represented by evidence that approximately 10% of newly budded daughter cells are [*psi*^−^], beginning immediately after Hsp104 induction (Ness et al. 2017). According to the trimming model, Hsp104 interacts with prion fibrils in two distinct modes: one which results in productive fragmentation and is thus required for stable propagation, and another in which it trims fibrils from the ends. Once fibrils are trimmed to a small enough size, the cores are subsequently degraded by the ubiquitin–protease system (Park et al. 2014). This model asserts that Hsp104 trims fibrils at wild-type expression level, but this activity is only significant during overexpression.

In Astor et al., we proposed a potential role for Apj1 in either model, based largely on Sahi et al.’s determination that Apj1 is involved in the degradation of sumoylated proteins (Sahi et al. 2013). In the case of the malpartitioning model, we posited that Apj1 may assist in malpartitioning, as cytoskeletal elements such as actin and tubulin are sumoylated (Panse et al. 2004; Castillo-Lluva et al. 2010; Gareau and Lima 2010). In the case of the trimming model, we suggested that Apj1 may be involved in assisting in or promoting the degradation of the prion cores. Sahi et al., found that single amino acid modifications to the ZBD and CTD I of Apj1 abolished Apj1’s function in the degradation of sumoylated proteins, but interestingly found the QS region dispensable (Sahi et al. 2013). Here, however, we showed that a construct of Apj1 comprised of only the J-domain and QS region is sufficient in carrying out its function in Hsp104-mediated curing of strong variants of [*PSI*^+^]. Likewise, we found that Apj1’s J-domain alone, as well as a construct expressing all of Apj1’s additional C-terminal domains, were both insufficient to support curing. Thus, the region of Apj1 that Sahi et al., determined to be critical in the degradation of sumoylated proteins is dispensable for Hsp104-mediated curing, and a region that Sahi et al., determined was dispensable for the degradation of sumoylated proteins is required for curing. Our new data clearly indicate that Apj1’s roles in Hsp104 overexpression curing of [*PSI*^+^] and the degradation of sumoylated proteins are mechanistically distinct. Taken together, our results strongly suggest a critical role of Apj1’s low-complexity QS region in Hsp104-mediated prion curing. In Astor et al. 2018, we demonstrated that by removing a portion of the low-complexity G/F-rich region of Sis1 (Fig. 2), the curing of strong variants of [*PSI*^+^] was completely abolished. Our results presented here in concert with the findings of Astor et al. suggest Hsp104-mediated curing of strong [*PSI*^+^] is critically dependent on at least two J-protein low-complexity regions.

What might the physiological role of Apj1’s QS region be? Some clues may exist in the literature. In one study, deletion of *APJ1* conferred mild sensitivity to caffeine, rapamycin, cycloheximide, calcofluor white, and wortmannin, which are significant effects considering Apj1’s normally very low expression level (Gillies et al. 2012). In another study, deletion of *APJ1* was found to impair the translocation of some GPI-anchored sequences into the ER, indicating an additional uncharacterized role in protein translocation (Ast et al. 2013) which remains to be further explored. It remains unclear, however, the extent to which these effects are due to the function of the QS region, specifically.

Apj1’s QS region, as its name implies, is not a typical J-protein glycine-rich region, in that it has notably high serine content and a Q-rich insert. Considering its low complexity and the failure of glycine-rich regions of other J-proteins to take on stable structures, it is likely that it may be an intrinsically disordered region (IDR), a functional region which is unlikely to take a defined three-dimensional structure (van der Lee et al. 2014). Intriguingly, the Q-rich IDR of Hsp42 was recently found to mediate its interaction with protein aggregates, which raises the question of whether IDRs of other chaperones, like Apj1, could act similarly (Grousl et al. 2018).

Another possibility is that the QS region is subject to a regulatory post-translational modification that is somehow critical for Hsp104-mediated curing. Indeed, bioinformatics studies of eukaryotic IDRs have suggested that these highly solvent-accessible regions are subject to significantly higher post-translational modifications than ordered protein regions (Xie et al. 2007; Tompa 2014; Faust et al. 2018). Although IDRs are generally thought to function based on the precise balance of, but not particular sequence of, positive, negative, polar, and hydrophobic residues (Faust et al. 2018), both Hsf1, the primary heat shock transcription factor in eukaryotes, and Pop2, the conserved exonuclease of the Ccr4-Not complex, were found to have phosphorylation events at specific serine residues within their respective IDRs that regulate their function (Guettouche et al. 2005; Yamamoto et al. 2008; Lien et al. 2019). Experimentally, Sis1’s G/F region, Ydj1’s G/F region, and Apj1’s QS region have already been found to completely lack phosphorylation and ubiquitination (Swaney et al. 2013). However, phosphorylation of serine residues can change dramatically in response to cell stress (Vlastaridis et al. 2017). It is plausible that phosphorylation was not detected in these three J-proteins’ IDRs simply because cells were grown under ideal conditions, and that phosphorylation may only be present under stressful conditions and/or when cells harbor [*PSI*^+^], a hypothesis that remains to be investigated. Regardless of the specific mechanism, our results, both here and in Astor et al., add to the body of evidence that underscores the integral, but poorly understood, roles of low-complexity regions of J-proteins in prion biology.

## References

[CR1] Aron R, Higurashi T, Sahi C, Craig EA (2007). J-protein co-chaperone Sis1 required for generation of [RNQ+] seeds necessary for prion propagation. EMBO J.

[CR2] Ast T, Cohen G, Schuldiner M (2013). A network of cytosolic factors targets SRP-independent proteins to the endoplasmic reticulum. Cell.

[CR3] Astor MT, Kamiya E, Sporn ZA (2018). Variant-specific and reciprocal Hsp40 functions in Hsp104-mediated prion elimination. Mol Microbiol.

[CR4] Bradley ME, Edskes HK, Hong JY (2002). Interactions among prions and prion “strains” in yeast. Proc Natl Acad Sci.

[CR5] Castillo-Lluva S, Tatham MH, Jones RC (2010). SUMOylation of the GTPase Rac1 is required for optimal cell migration. Nat Cell Biol.

[CR6] Chernoff YO, Lindquist SL, Ono B (1995). Role of the chaperone protein Hsp104 in propagation of the yeast prion-like factor [PSI+]. Science.

[CR7] Cox B, Tuite M (2018). The life of [PSI]. Curr Genet.

[CR8] Cox B, Ness F, Tuite M (2003). Analysis of the generation and segregation of propagons: entities that propagate the [PSI+] prion in yeast. Genetics.

[CR9] Craig EA, Marszalek J (2017). How do J-proteins get Hsp70 to do so many different things?. Trends Biochem Sci.

[CR10] Derkatch IL, Chernoff YO, Kushnirov VV (1996). Genesis and variability of [PSI] prion factors in *Saccharomyces cerevisiae*. Genetics.

[CR11] Derkatch IL, Bradley ME, Zhou P (1997). Genetic and environmental factors affecting the de novo appearance of the [PSI+] prion in Saccharomyces cerevisiae. Genetics.

[CR12] Du Z, Park KW, Yu H (2008). Newly identified prion linked to the chromatin-remodeling factor Swi1 in *Saccharomyces cerevisiae*. Nat Genet.

[CR13] Faust O, Grunhaus D, Shimshon O (2018). Protein regulation by intrinsically disordered regions: a role for subdomains in the IDR of the HIV-1 Rev protein. ChemBioChem.

[CR14] Gareau JR, Lima CD (2010). The SUMO pathway: emerging mechanisms that shape specificity, conjugation and recognition. Nat Rev Mol Cell Biol.

[CR15] Ghaemmaghami S, Huh W-K, Bower K (2003). Global analysis of protein expression in yeast. Nature.

[CR16] Gillies AT, Taylor R, Gestwicki JE (2012). Synthetic lethal interactions in yeast reveal functional roles of J protein co-chaperones. Mol BioSyst.

[CR17] Greene LE, Zhao X, Eisenberg E (2018). Curing of [PSI+] by Hsp104 overexpression: clues to solving the puzzle. Prion.

[CR18] Grousl T, Ungelenk S, Miller S (2018). A prion-like domain in Hsp42 drives chaperonefacilitated aggregation of misfolded proteins. J Cell Biol.

[CR19] Guettouche T, Boellmann F, Lane WS, Voellmy R (2005). Analysis of phosphorylation of human heat shock factor 1 in cells experiencing a stress. BMC Biochem.

[CR20] Harris JM, Nguyen PP, Patel MJ (2014). Functional diversification of Hsp40: distinct J-protein functional requirements for two prions allow for chaperone-dependent prion selection. PLoS Genet.

[CR21] Haslberger T, Zdanowicz A, Brand I (2008). Protein disaggregation by the AAA+ chaperone ClpB involves partial threading of looped polypeptide segments. Nat Struct Mol Biol.

[CR22] Helsen CW, Glover JR (2012). A new perspective on Hsp104-mediated propagation and curing of the yeast prion [PSI+]. Prion.

[CR23] Higurashi T, Hines JK, Sahi C (2008). Specificity of the J-protein Sis1 in the propagation of 3 yeast prions. Proc Natl Acad Sci.

[CR24] Hines JK, Craig EA (2011). The sensitive [SWI+] prion. Prion.

[CR25] Hines JK, Higurashi T, Srinivasan M, Craig EA (2011). Influence of prion variant and yeast strain variation on prion-molecular chaperone requirements. Prion.

[CR26] Hines JK, Li X, Du Z (2011). [SWI+], the prion formed by the chromatin remodeling factor Swi1, is highly sensitive to alterations in hsp70 chaperone system activity. PLoS Genet.

[CR27] Holmes DL, Lancaster AK, Lindquist S, Halfmann R (2013). Heritable remodeling of yeast multicellularity by an environmentally responsive prion. Cell.

[CR28] Jung G, Jones G, Wegrzyn RD, Masison DC (2000). A role for cytosolic hsp70 in yeast [PSI(+)] prion propagation and [PSI(+)] as a cellular stress. Genetics.

[CR29] Kampinga HH, Craig EA (2010). The HSP70 chaperone machinery: J proteins as drivers of functional specificity. Nat Rev Mol Cell Biol.

[CR30] Killian AN, Hines JK (2018). Chaperone functional specificity promotes yeast prion diversity. PLoS Pathog.

[CR31] Killian AN, Miller SC, Hines JK (2019). Impact of amyloid polymorphism on prion-chaperone interactions in yeast. Viruses.

[CR32] Kondrashkina AM, Antonets KS, Galkin AP, Nizhnikov AA (2014). Prion-like determinant [NSI +] decreases the expression of the SUP45 gene in *Saccharomyces cerevisiae*. Mol Biol.

[CR33] Kryndushkin DS, Smirnov VN, Ter-Avanesyan MD, Kushnirov VV (2002). Increased expression of Hsp40 chaperones, transcriptional factors, and ribosomal protein Rpp0 can cure yeast prions. J Biol Chem.

[CR34] Kryndushkin DS, Alexandrov IM, Ter-Avanesyan MD, Kushnirov VV (2003). Yeast [PSI+] prion aggregates are formed by small Sup35 polymers fragmented by Hsp104. J Biol Chem.

[CR35] Kryndushkin DS, Engel A, Edskes H, Wickner RB (2011). Molecular chaperone Hsp104 can promote yeast prion generation. Genetics.

[CR36] Li J, Qian X, Sha B (2009). Heat shock protein 40: structural studies and their functional implications. Protein Pept Lett.

[CR37] Lian HY, Zhang H, Zhang ZR (2007). Hsp40 interacts directly with the native state of the yeast prion protein Ure2 and inhibits formation of amyloid-like fibrils. J Biol Chem.

[CR38] Liebman SW, Chernoff YO (2012). Prions in yeast. Genetics.

[CR39] Lien PTK, Viet NTM, Mizuno T (2019). Pop2 phosphorylation at S39 contributes to the glucose repression of stress response genes, HSP12 and HSP26. PLoS One.

[CR40] Lopez N, Aron R, Craig EA (2003). Specificity of class II Hsp40 Sis1 in maintenance of yeast prion [RNQ +]. Mol Biol Cell.

[CR41] Luke MM, Sutton A, Arndt KT (1991). Characterization of SIS1, a *Saccharomyces cerevisiae* homologue of bacterial dnaJ proteins. J Cell Biol.

[CR42] Matveenko AG, Barbitoff YA, Jay-Garcia LM (2018). Differential effects of chaperones on yeast prions: current view. Curr Genet.

[CR43] Moriyama H, Edskes HK, Wickner RB (2000). [URE3] Prion propagation in *Saccharomyces cerevisiae*: requirement for chaperone Hsp104 and curing by overexpressed chaperone Ydj1p. Mol Cell Biol.

[CR44] Ness F, Cox BS, Wongwigkarn J (2017). Over-expression of the molecular chaperone Hsp104 in *Saccharomyces cerevisiae* results in the malpartition of [PSI+] propagons. Mol Microbiol.

[CR45] Oliver EE, Troisi EM, Hines JK (2017). Prion-specific Hsp40 function: the role of the auxilin homolog Swa2. Prion.

[CR46] Panse VG, Hardeland U, Werner T (2004). A proteome-wide approach identifies sumoylated substrate proteins in yeast. J Biol Chem.

[CR47] Park YN, Zhao X, Yim YI (2014). Hsp104 overexpression cures *Saccharomyces cerevisiae* [PS +] by causing dissolution of the prion seeds. Eukaryot Cell.

[CR48] Patel BK, Gavin-Smyth J, Liebman SW (2009). The yeast global transcriptional co-repressor protein Cyc8 can propagate as a prion. Nat Cell Biol.

[CR49] Prusiner SB (2013). Biology and genetics of prions causing neurodegeneration. Annu Rev Genet.

[CR50] Sahi C, Craig EA (2007). Network of general and specialty J protein chaperones of the yeast cytosol. Proc Natl Acad Sci USA.

[CR51] Sahi C, Kominek J, Ziegelhoffer T (2013). Sequential duplications of an ancient member of the DnaJ-family expanded the functional chaperone network in the eukaryotic cytosol. Mol Biol Evol.

[CR52] Saifitdinova AF, Nizhnikov AA, Lada AG (2010). [NSI+]: a novel non-Mendelian nonsense suppressor determinant in *Saccharomyces cerevisiae*. Curr Genet.

[CR53] Schilke BA, Ciesielski SJ, Ziegelhoffer T (2017). Broadening the functionality of a J-protein/Hsp70 molecular chaperone system. PLoS Genet.

[CR54] Simpson-Lavy K, Kupiec M (2018). A reversible liquid drop aggregation controls glucose response in yeast. Curr Genet.

[CR55] Sondheimer N, Lopez N, Craig EA, Lindquist S (2001). The role of Sis1 in the maintenance of the [RNQ+] prion. EMBO J.

[CR56] Sporn ZA, Hines JK (2015). Hsp40 function in yeast prion propagation: amyloid diversity necessitates chaperone functional complexity. Prion.

[CR57] Stein KC, True HL (2014). Extensive diversity of prion strains is defined by differential chaperone interactions and distinct amyloidogenic regions. PLoS Genet.

[CR58] Swaney DL, Beltrao P, Starita L (2013). Global analysis of phosphorylation and ubiquitylation cross-talk in protein degradation. Nat Methods.

[CR59] Tanaka M, Collins SR, Toyama BH, Weissman JS (2006). The physical basis of how prion conformations determine strain phenotypes. Nature.

[CR60] Tipton KA, Verges KJ, Weissman JS (2008). In vivo monitoring of the prion replication cycle reveals a critical role for Sis1 in delivering substrates to Hsp104. Mol Cell.

[CR61] Tompa P (2014). Multisteric regulation by structural disorder in modular signaling proteins: an extension of the concept of allostery. Chem Rev.

[CR62] Troisi EM, Rockman ME, Nguyen PP (2015). Swa2, the yeast homolog of mammalian auxilin, is specifically required for the propagation of the prion variant [URE3-1]. Mol Microbiol.

[CR63] Tsai J, Douglas MG (1996). A Conserved HPD sequence of the J-domain is necessary for YDJ1 stimulation of Hsp70 ATPase activity at a site distinct from substrate binding. J Biol Chem.

[CR64] Tuite MF, Koloteva-Levin N (2004). Propagating prions in fungi and mammals. Mol Cell.

[CR65] van der Lee R, Buljan M, Lang B (2014). Classification of intrinsically disordered regions and proteins. Chem Rev.

[CR66] Verma KD, Massing JO, Kamper SG (2017). Synthesis and evaluation of MR probes for targeted-reporter imaging. Chem Sci.

[CR67] Vlastaridis Panayotis, Papakyriakou Athanasios, Chaliotis Anargyros, Stratikos Efstratios, Oliver Stephen G., Amoutzias Grigorios D. (2017). The Pivotal Role of Protein Phosphorylation in the Control of Yeast Central Metabolism. G3: Genes|Genomes|Genetics.

[CR68] Volkov KV, Aksenova AY, Soom MJ (2002). Novel non-mendelian determinant involved in the control of translation accuracy in *Saccharomyces cerevisiae*. Genetics.

[CR69] Wendler P, Shorter J, Snead D (2009). Motor mechanism for protein threading through Hsp104. Mol Cell.

[CR70] Wickner RB, Shewmaker FP, Bateman DA (2015). Yeast prions: structure, biology, and prion-handling systems. Microbiol Mol Biol Rev.

[CR71] Wickner RB, Bezsonov EE, Son M (2018). Anti-prion systems in yeast and inositol polyphosphates. Biochemistry.

[CR72] Winkler J, Tyedmers J, Bukau B, Mogk A (2012). Hsp70 targets Hsp100 chaperones to substrates for protein disaggregation and prion fragmentation. J Cell Biol.

[CR73] Xie H, Vucetic S, Iakoucheva LM (2007). Functional anthology of intrinsic disorder. 3. Ligands, post-translational modifications, and diseases associated with intrinsically disordered proteins. J Proteome Res.

[CR74] Yamamoto N, Maeda Y, Ikeda A, Sakurai H (2008). Regulation of thermotolerance by stress-induced transcription factors in *Saccharomyces cerevisiae*. Eukaryot Cell.

[CR75] Yokom AL, Gates S, Jackrel ME (2017). Spiral architecture of the Hsp104 disaggregase reveals the structural basis for polypeptide translocation. Nature Struct Mol biol.

[CR76] Zhao X, Rodriguez R, Silberman RE (2017). Heat shock protein 104 (Hsp104)-mediated curing of [PSI+] yeast prions depends on both [PSI+] conformation and the properties of the Hsp104 homologs. J Biol Chem.

